# Characterization and Comparative Analysis of Whole-Transcriptome Sequencing in High- and Low-Fecundity Chongming White Goat Ovaries during the Estrus Phase

**DOI:** 10.3390/ani14070988

**Published:** 2024-03-22

**Authors:** Yuexia Lin, Lingwei Sun, Jianjun Dai, Yuhua Lv, Rongrong Liao, Xiaohui Shen, Jun Gao

**Affiliations:** 1 Institute of Animal Husbandry and Veterinary Science, Shanghai Academy of Agricultural Sciences, Shanghai 201106, China; lyxia05@163.com (Y.L.); sunlingwei1987@126.com (L.S.); daijianjun@saas.sh.cn (J.D.); 18918162132@163.com (Y.L.); lrrnd@163.com (R.L.); 2 Division of Animal Genetic Engineering, Shanghai Municipal Key Laboratory of Agri-Genetics and Breeding, Shanghai 201106, China; 3 Key Laboratory of Livestock and Poultry Resources (Pig) Evaluation and Utilization, Ministry of Agriculture and Rural Affairs, Shanghai 201106, China

**Keywords:** goat, ovary, fecundity, non-coding RNAs, ceRNA

## Abstract

**Simple Summary:**

Optimal reproductive performance on goat farms is an important trait in terms of production and economics. From a genetic perspective, understanding molecular mechanisms through the use of RNA-seq technology can help us better understand goat herds. In this study, a whole-transcriptome sequencing approach was used to identify lncRNA, circRNA, miRNA, and mRNA expression in the ovaries of Chongming white goats in terms of high and low fecundity during the estrus phase. These results were helpful in terms of further studying the molecular mechanisms of goat reproduction.

**Abstract:**

Reproductive performance is one of the most important economic traits in the goat industry. Increasing the number of goats is an effective measure to improve production efficiency and reduce production costs. Ovaries are important reproductive organs in female mammals that directly affect the estrous cycle and reproductive abilities. Understanding the complex transcription network of non-coding RNAs (lncRNAs, circRNAs, and miRNAs) and messenger RNA (mRNA) could lead to significant insights into the ovarian regulation of the reproductive processes of animals. However, the whole-transcriptome analysis of the non-coding RNAs and mRNA of the ovaries in Chongming white goats between high-fecundity (HP) and low-fecundity (LP) groups is limited. In this study, a whole-transcriptome sequencing approach was used to identify lncRNA, circRNA, miRNA, and mRNA expression in the ovaries of Chongming white goats during the estrus phase using RNA-Seq technology. More than 20,000 messenger RNAs (mRNAs), 10,000 long non-coding RNAs (lncRNAs), 3500 circular RNAs (circRNAs), and 1000 micro RNAs (miRNAs) were identified. A total of 1024 differential transcripts (724 mRNAs, 112 lncRNAs, 178 circRNAs, and 10 miRNAs) existing between the HP and the LP groups were revealed through a bioinformatics analysis. They were enriched in the prolactin signaling pathway, the Jak–STAT signaling pathway, and the GnRH signaling pathway, as well as various metabolic pathways. Differentially expressed mRNAs (such as *LYPD6*, *VEGFA*, *NOS3*, *TNXB*, and *EPHA2*) and miRNAs (such as *miR-10a-5p*) play key roles in the regulation of goat ovaries during the estrus phase. The enrichment of pathways related to reproduction, such as the Hippo, Hedgehog, PI3K–AKT, and MAPK signaling pathways, suggests that they might be involved in the prolificacy of goat ovaries. Overall, we identified several gene modules associated with goat fecundity and provided a basis for a molecular mechanism in the ovaries of Chongming white goats.

## 1. Introduction

Chongming white goats, a unique indigenous Chinese goat breed, are farmed to provide milk, meat, and fiber, and also provide other benefits. The optimal reproductive performance of goats is an important production and economic trait affecting the reproductive performance of farms [1]. Accordingly, uncovering the molecular mechanisms involved may help our understanding of multiple goats in terms of population genetics. Moreover, the prolificacy of goats and sheep has been of interest in recent decades. To date, bone morphogenetic protein receptor type 1B (BMPR1B), bone morphogenetic protein 15 (BMP15), growth differentiation factor 9 (GDF9), and the X-linked maternally imprinted gene (FecX2) have been identified as major genes for high-fecundity traits in some sheep breeds [2]. However, there are relatively few studies on the reproductive performance of goats.

As the gonadal organs of female mammals, ovaries play an important role in generating mature oocytes and producing hormones that regulate reproductive functions, which is very closely related to litter size [3,4]. Consequently, it is essential to elucidate the underlying molecular mechanisms associated with prolificacy and explore effective breeding measures that could improve the regularity of fetal litter size in Chongming goats. The molecular mechanisms of epigenetics include DNA methylation, non-coding RNAs (ncRNAs), histone variants, and their modifications [5]. At present, the characterization of ncRNAs, such as microRNA (miRNA), long non-coding RNA (lncRNA), and circular RNA (circRNA), plays a more regulatory role in various cell functions and has become one of the most fruitful areas of biological research in animals [6]. Among them, circRNAs, covalently closed loops formed via a non-canonical splicing method known as back-splicing, have increasingly been shown to regulate various physiological and developmental processes by acting as a sponge for miRNAs or RNA-binding proteins (RBPs) [7]. Due to base pairing, miRNAs are short (approximately 22 nt) endogenous ncRNAs that govern gene expression by degrading mRNA or by inhibiting mRNA translation; lncRNAs, another class of ncRNA that are more than 200 nt in length, can interact with DNA, RNA, and proteins for chromatin modification, transcription, and post-transcriptional regulation [8]. With the progress of deep-sequencing ncRNA technology and bioinformatics in recent years, research investigating the epigenetic alterations of ncRNAs has become prominent in the field of goat fecundity research. The integrated analysis of the miRNA and mRNA expression profiles of goat ovaries in Chuanzhong black goats between high- and low-fecundity goats showed that several candidate genes, including miR-182, miR-122, and miR-206, were involved in goat fecundity [9]. Additionally, a recent study shed some light on the role of several lncRNAs in the regulation of follicle development in the ovaries of Chuanzhong black goats [10]. Further, *chi_circ_0008219* in the ovarian follicles of Macheng black goats was found to be involved in a circRNA–miRNA–mRNA co-expression network and was observed to sponge three ovarian follicle-related miRNAs [11]. For high-yield traits in goats, researchers found that chi-miR-439-3p-JAK3 was a novel regulatory pathway in the primary granulosa cells of ovarian tissues via a whole-genome expression analysis and found that it might play key functional roles in oogenesis [12]. Another study analyzed the circRNA profiles of goat ovarian tissues among high- and low-fecundity goats and found that miR-133a-3p, miR-133b, miR-129-3p, and miR-21 had significant effects on the reproductive traits of goats [13].

In recent years, many goat breeds have been investigated through the use of sequencing, providing resources for studies on goat fertility. However, because of the tissue- and condition-specific natures of most ncRNAs and mRNAs and low-sequence conservation among species [14,15], the ncRNA and mRNA expression profiles of the ovaries of Chongming white goats between high- and low-fecundity groups are still poorly understood. In this study, a whole-transcriptome sequencing approach was used to identify lncRNA, circRNA, miRNA, and mRNA expression in the ovaries of Chongming white goats during the estrus phase. Then, the differentially expressed ones were identified to investigate the biological functions of the enriched pathways, and lncRNA–miRNA–mRNA and circRNA–miRNA–mRNA networks were constructed to reveal novel insights into the regulation of multi-lamb traits in goats.

## 2. Materials and Methods

### 2.1. Ethics Statement

All of the study protocols were approved by the ethics committee for the care and use of laboratory animals at the Shanghai Academy of Agricultural Sciences, China (permit no.: SAASPZ0522048). All of the experiments were performed in accordance with the relevant guidelines and regulations set by the Ministry of Agriculture of the People’s Republic of China.

### 2.2. Animals and Sample Preparation

Chongming white goats were raised at the Chongming white goat test station of the Shanghai Academy of Agricultural Sciences. According to the reproduction records, age, and body size, a total of six healthy female goats (age: 3.6–4.8 years) were used in this study from two groups: the high-prolificacy group (HP: *n* = 3) and low-prolificacy group (LP: *n* = 3). In this study, all of the goat samples had three parity records relating to litter size. The goats in the LP group lambed only one kid per litter, and the goats in the HP group lambed no less than two kids per litter. There were no differences in the animal’s environment or diet. The animals were deprived of food overnight before they were slaughtered.

All of the selected goats were synchronized to estrus using a progesterone vaginal suppository (CIDR) [13]. After 16 days of the synchronous estrus treatment, the vaginal suppository was removed. The experimental goats were checked for signs of estrus three times per day. The goats were considered to be in estrus when they allowed a male goat to mount. The selected goats were slaughtered within 24 h of estrus, and both whole ovaries from each goat were collected immediately. Then, the tissue samples were frozen in liquid nitrogen, transported to the lab, and stored at −80 °C until use. The total RNA was isolated from the ovaries using the RNAiso Plus reagent (Takara, Tokyo, Japan) according to the manufacturer’s instructions.

### 2.3. miRNA Sequencing

Each sample was divided into differently sized segments via polyacrylamide gel electrophoresis (PAGE), and a portion of the gel corresponding to 18–30 nucleotides was cut to be linked with 3′adaptors. Further purification was achieved using denaturing Urea-PAGE gel to obtain segments of 36–44 nucleotides, which were then ligated with 5′adaptors to obtain the miRNA. Next, reverse-transcription PCR was carried out, resulting in segments of 140–160 base pairs that were separated using 3.5% agarose gel. A miRNA library for miRNA sequencing was constructed using Illumina HiSeqTM 4000 sequencing (Illumina Inc., San Diego, CA, USA).

After sequencing, the FASTX toolkit (FASTX-toolkit 0.0.14, http://hannonlab.cshl.edu/fastx_toolkit/, accessed on 19 June 2022.) and FastQC were used to remove the adapter sequences and low-quality sequences, respectively. The obtained tag sequences of the small RNA were aligned using Sanger miRBase (*Capra hircus*), piRNA, ncRNA, and Rfam databases using blastn with standard parameters to identify miRNA and to remove rRNAs, scRNAs, snoRNAs, snRNAs, and tRNAs. Novel miRNAs were identified by predicting the hairpin structure using the miRCat program with default parameters. Differentially expressed miRNAs (DEmiRNAs) were identified with *p*-values of <0.05 and |log2 (fold change)| ≥1. Furthermore, miRNAs with log2 values >1 were labeled as upregulated genes (up), and those with values of <−1 were labeled as downregulated genes (down). Gene ontology (GO) term enrichment and the Kyoto Encyclopedia of Genes and Genomes (KEGG) pathway enrichment analyses of target genes of DEmiRNAs were performed using DAVID bioinformatic resources (v6.8).

### 2.4. circRNA Sequencing

To enrich the circular RNA, the total RNA was treated with an RNAse R kit (Epicentre Biotechnologies, Madison, WI, USA) to selectively remove the linear RNA. The remaining RNA was used to construct a circRNA sequencing library. Briefly, the target RNAs were broken into short (approximately 200–500 nucleotide) fragments using a fragmentation buffer. Then, the cDNAs were synthesized using the RNA fragments as templates for the N6 random primer. Second-strand cDNA was subsequently synthesized by adding the dNTP mix, RNase H, DNA polymerase I, and buffer. The DNA was column-purified (QIAquick PCR Purification Kit) and eluted in 40 μL of EB buffer. Following the PCR amplification, the obtained circRNA library was subjected to Illumina HiSeq 4000 (150-bp paired-end reads; Illumina, United States). The raw reads were cleaned by removing reads with adapters, unknown nucleotides >5%, and low-quality reads (<Q20), which were compared with the reference genome. Unmapped reads were intercepted at both ends as an anchor. In order to analyze the sequencing data that could not be aligned to the reference genome directly, we used Find_circ (v1.0) and CIRCexplorer2 to identify circRNAs [16]. A RPM (reads per million mapping) method was applied to calculate the relative expression abundance of the circRNAs. Differentially expressed circRNAs (DEcircRNAs) were identified with *p*-values of <0.05 and |log2 (fold change)| ≥1. The functional annotation of the circRNAs was based on annotations from the GO analysis and KEGG pathway enrichment analysis.

### 2.5. mRNA and lncRNA Sequencing

First, the ribosomal RNA was removed from the total RNA to maximize the retention of all non-coding RNAs. The enriched mRNA was then fragmented into short fragments of approximately 200 nucleotides, which acted as templates for the cDNA synthesis. After fragmentation, first-strand cDNA was synthesized using random hexamer-primed reverse transcription, followed by second-strand cDNA synthesis using the dNTP mix, RNase H, DNA polymerase I, and buffer, and then transcribed via PCR amplification to establish the library used for sequencing. The clean data containing high-quality reads were obtained from the raw reads after filtering the reads with an adapter and against an N content greater than 10% and low-quality reads (Q ≤ 20). The processed mRNA reads were mapped to the reference genome to build the mRNA library and calculate the expression of mRNAs. The RPKM (reads per kilobase transcriptome per million mapped reads) method was used to normalize the gene expression. Genes with RPKM  <  1 were removed as non-expressed genes. By controlling the false discovery rate (FDR), the *p*-value threshold for multiple tests could be determined. Differentially expressed mRNAs (DEmRNAs) were identified using the DESeq R package (www.huber.embl.de/users/anders/DESeq/) with an adjusted *p*-value of ≤0.05 and log2 (fold change)| ≥0.6). Meanwhile, the GO function and KEGG pathway enrichment analysis were performed. The candidate lncRNAs from the mRNA were further screened through the use of the Coding Potential Calculator (CPC) and the Coding–Non-Coding Index (CNCI) by evaluating the coding ability.

### 2.6. Gene Co-Expression Analysis

To predict mRNAs that interacted with miRNAs and mRNAs, MiRanda software (version 3.3a) and online miRNA target prediction software (http://www.microrna.org/microrna/home.do, accessed on 26 December 2022) were used. Then, two methods were used to investigate the intersection of the miRNA target genes to predict the forecasting results of the target genes. Spearman’s rank correlation coefficient between DEmRNAs and DEmiRNAs was tested using the cor.test function in R, with a correlation coefficient of cor < 0. Additionally, the MiRanda program was also employed to predict the interaction between lncRNA and miRNA and the target relationship between the lncRNA and mRNA (cis and trans). The co-expression analysis and target relationship analysis were combined to produce the final results, and the cor.test function was used to calculate the correlations. For the screening of the differences between mRNA and lncRNA expression, the standards were cor ≠ 0 and a *p*-value of <0.05. Based on the positive correlation between mRNA and the lncRNA (cor > 0 and *p*-value < 0.05), a lncRNA–miRNA–mRNA network was constructed, and the network was visualized using Cytoscape software (version 3.6.1) based on the ceRNA theory [17].

For the following procedure, the same process as that detailed above was used. MiRanda and RNAhybird (version 2.1.2) were used to predict the target relationships between all miRNAs and differential mRNA and differential circRNA. Spearman’s rank correlation was performed using the cor.test function. Based on the positive correlation between circRNAs and mRNAs (cor > 0 and *p*-value < 0.05), a circRNA–miRNA–mRNA network was constructed and the network was visualized.

### 2.7. Validation of RNA-Seq Results Analyzed Using Quantitative PCR (qPCR)

After the RNA extraction, the reverse transcription of total RNA was performed using PrimeScript™ Reverse Transcriptase (TaKaRa, Dalian, China). To validate the RNA sequencing results, qRT-PCR was performed to analyze the expression of fourteen selected mRNAs. The primers for qRT-PCR were designed using Primer Premier (version 5.0) software (PREMIER Biosoft) and their specificity was verified through the use of PCR (Table 1). *RPL19* (*XM_005693740.3*) was used as the endogenous control for the mRNA expression analysis [18]. For the qRT-PCR analysis, each reaction was performed with three biological and three technical replicates. Following the procedures suggested by the manufacturer, the qRT-PCR analysis was conducted using a StepOne/StepOnePlus™ real-time PCR system (Applied Biosystems, Foster City, CA, USA) and SYBR Green qPCR Mix (Applied Biosystems). The final volume of the qRT-PCR reaction system was 20 µL: 10 µL of SYBR Premix Ex Taq II, 1 µL of forward primer (0.5 µM), 1 µL of reverse primer (0.5 µM), 1 µL of cDNA, and 7 µL of ddH_2_O. The conditions for qRT-PCR were as follows: initial denaturation at 95 °C for 5 min, followed by 40 cycles of 60 °C for 30 s, 54~60 °C for 60 s, and 95 °C for 15 s. The melting curve parameters were as follows: 65 °C to 95 °C with increments of 0.5 °C/s. The relative expressions of the mRNAs were calculated using the 2−ΔΔCq method [19]. Statistical analyses were performed with SPSS (version 20.0; Chicago, IL, USA) and the figures were prepared using GraphPad Prism (version 8.0; GraphPad Software, San Diego, CA, USA). A one-way analysis of variance (ANOVA) was used for comparison, where a *p*-value of < 0.05 was considered statistically significant.

## 3. Results

### 3.1. Overview of circRNA Sequencing

**Figure 1:**
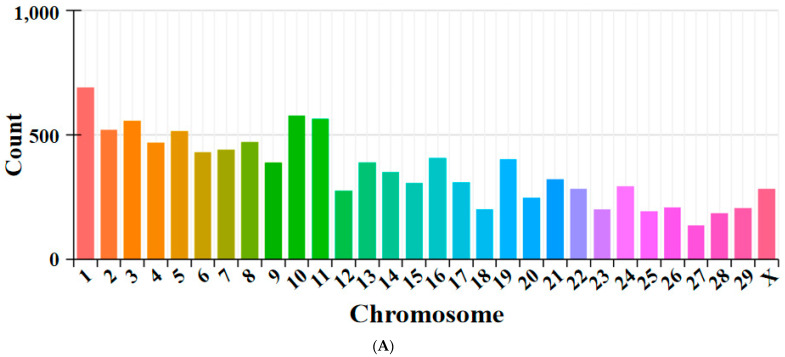

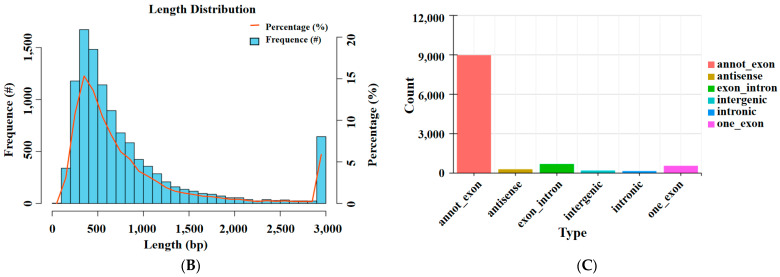
Genomic features of the circRNAs identified in the goats. (**A**) The distributions of circRNAs across different chromosomes. (**B**) The length distribution density of circRNAs. (**C**) The number of circRNA types.

An average of 77,670,277 (LP) and 81,049,125 (HP) clean reads were obtained across two libraries. In total, 99.82% (LP) and 99.80% (HP) high-quality (HQ) clean reads were generated after the data were trimmed and the quality filters were applied. HQ clean reads were first stripped of mapped rRNA reads and then 20 base pairs at both ends of the unmapped reads were chosen for further alignment to the caprine genome. In total, 10,907 novel circRNAs were identified in this study (Supplementary Table S1), which were named and numbered from novel_circ_000001 to novel_circ_010908. A detailed description was provided, including their gene ID, NCBI ID, chromosomal location, start and end sites, length, and circRNA types. The distributions of the identified circRNAs in different chromosomes are shown in Figure 1A. These circRNAs were distributed on all chromosomes, including 29 autosomes and the X chromosome, but mainly existed in chromosomes 1, 2, 3, 4, 10, and 11. The majority of the circRNAs were 300–400 nt in length (Figure 1B), though some were greater than 3000 bp. Furthermore, the most common type of circRNA was annot_exon.

### 3.2. CircRNA Source Gene Analysis and Differentially Expressed DEcircRNAs Analysis

**Figure 2:**
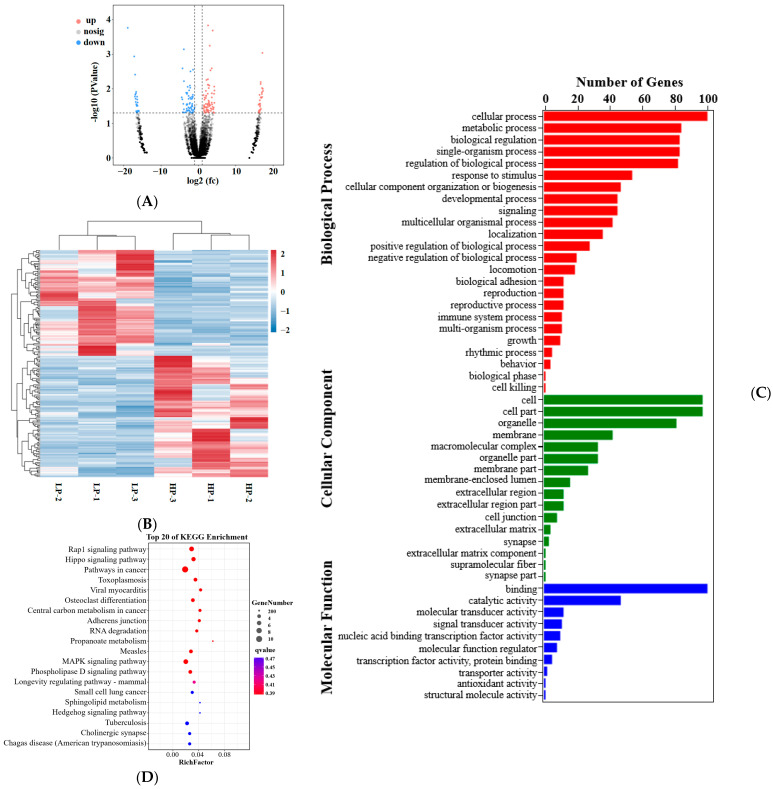
Differentially expressed circRNAs and the functional annotation analysis of circRNA source genes. (**A**) Scatter plots of differentially expressed circRNAs. Red, blue, and gray dots in the graph represent transcripts that were significantly upregulated, downregulated, or had non-significant differences, respectively. (**B**) Heatmap analysis of the differentially expressed circRNAs in the LP and HP groups. (**C**) Bar plot of the 50 enriched GO terms for differentially expressed circRNA source genes. (**D**) Scatterplot of top 20 pathways in KEGG enrichment.

Compared with the LP group, 178 DEcircRNAs were identified in the HP group, 95 of which were upregulated and 83 downregulated (Figure 2A; further details of these circRNAs are available in Supplementary Table S2). A heatmap of the DEcircRNAs is presented in Figure 2B. To further explore the potential functions of DEcircRNAs, the GO enrichment and KEGG pathway analyses were carried out on their target genes. Figure 2C shows the enriched GO terms for differentially expressed circRNA source genes in three GO ontologies (Supplementary Table S3): biological processes (847 genes), cellular component (468 genes), and molecular function (197 genes). Furthermore, the highly enriched terms in the GO enrichment were related to the regulation of biological processes, biological regulation, metabolic processes, cellular processes, single-organism processes, binding, cell, cell part, and organelles. In order to analyze the source genes associated with intracellular metabolic processes, 184 KEGG pathways were identified (Supplementary Table S4). The top 20 enriched pathways are displayed in Figure 2D. Of those, the Rap1 signaling pathway, Hippo signaling pathway, Hedgehog signaling pathway, MAPK signaling pathway, and phospholipase D signaling pathway were closely related to reproduction traits.

### 3.3. Sequencing and DEmiRNAs Analysis

**Figure 3:**
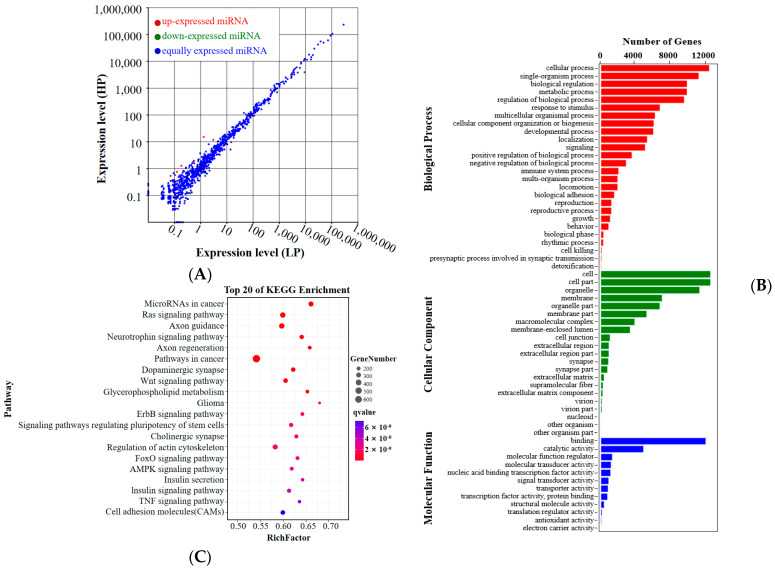
Differentially expressed miRNAs and the functional annotation analysis of target genes of differentially expressed miRNAs. (**A**) Scatter plots of differentially expressed miRNAs. Red, green, and blue dots in the graph represent transcripts that were significantly upregulated, downregulated, or that had non-significant differences, respectively. (**B**) Bar plot of the 57 enriched GO terms for target genes of differentially expressed miRNAs. (**C**) Scatterplot of top 20 pathways in KEGG enrichment.

After the removal of adapter sequences and low-quality reads, an average of 12, 494, and 240 (98.39% of raw reads) and 13, 409, and 301 (98.36% of raw reads) clean tags were obtained from the three samples of the LP group and three samples of the HP group, respectively. Approximately 76.90% and 76.06% of the total clean reads from the LF and HF groups were mapped to the reference database, respectively. By combining the conserved and novel miRNAs, 901 known miRNAs and 71 novel miRNAs were identified, and the expression of the miRNAs is listed in Supplementary Table S5. Compared with the LP group, ten DEmiRNAs were identified in the HP group, six of which were upregulated and four were downregulated (Figure 3A; further details of these miRNAs are available in Supplementary Table S6). After that, the target genes of DEmiRNAs were analyzed for GO and KEGG pathway enrichment. As a result of the GO enrichment analysis, target genes were found to be involved in 59 GO terms (Figure 3B). Of these, 26 GO terms were assigned to biological processes, 21 GO terms were assigned to cellular components, and 12 GO terms were assigned to molecular functions (Supplementary Table S7). The highly enriched terms in the GO enrichment were cellular processes, metabolic processes, biological regulation, single-organism processes, the regulation of biological processes, cell, cell part, organelles, binding, and catalytic activity. As a result of the KEGG enrichment analysis, 351 pathways were found to be associated with the target genes (Supplementary Table S8). The top 20 enriched pathways are displayed in Figure 3C. Of those, the RAS signaling pathway, Wnt signaling pathway, glycerophospholipid metabolism, ErbB signaling pathway, AMPK signaling pathway, and cell adhesion molecules (CAMs) were closely related to reproduction traits.

### 3.4. Sequencing and DEmRNA and DElncRNA Analysis

**Figure 4:**
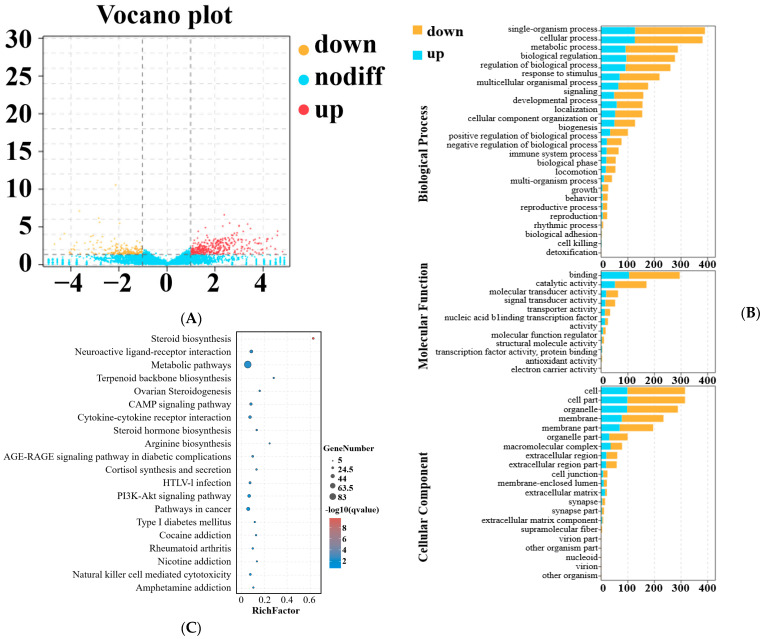
Differential mRNA expression analysis between LP and HP groups. (**A**) Scatter plots of differentially expressed mRNA expression. Red, blue, and gray dots in the graph represent transcripts that were significantly upregulated, downregulated, or those that had non-significant differences, respectively. (**B**) Bar plot of the 59 enriched GO terms for target genes of differentially expressed mRNAs. (**C**) Scatterplot of top 20 pathways in KEGG enrichment.

After the removal of rRNA and low-quality reads, we obtained an average of 81,049,125 (99.82% of raw reads) and 77,670,277 HQ clean reads (99.8% of raw reads) from the LP and HP groups, respectively. Of the HQ clean reads, on average, 93.84% and 93.91% of the reads from the LP and HP groups were uniquely mapped to the goat reference genome, respectively. Here, we identified 20, 743 known mRNAs and 10 novel mRNAs. A total of 724 DEmRNAs (477 up- and 724 downregulated) were identified in the HP group compared to the LP group (Figure 4A). For a detailed list of all these mRNAs and their expression levels, see Supplementary Table S9. We also analyzed the DEmRNAs based on KEGG pathways and GO terms. As a result of the GO enrichment analysis, genes were found to be involved in 57 GO terms (Figure 4B). Of these, 25 GO terms were involved in biological processes, 11 GO terms were involved in molecular functions, and 21 GO terms were involved in cellular components (Supplementary Table S10). The highly enriched terms in the GO enrichment were single-organism processes, cellular processes, binding, cell, and cell part. DEmRNAs could be enriched into 301 KEGG pathways (Supplementary Table S11), and the top 20 enriched pathways are shown in Figure 4C. Of those, steroid biosynthesis, ovarian steroidogenesis, steroid hormone biosynthesis, and cortisol synthesis and secretion were closely related to reproduction traits. The CAMP signaling pathway and PI3K–AKT signaling pathway were also significantly enriched.

We used CPC2 (Coding Potential Calculator 2) and CNCI (Coding–Non-Coding Index) to identify lncRNAs from 5894 transcripts with non-coding potential and identified 3595 lncRNAs. A total of 112 DElncRNAs (54 upregulated and 58 downregulated) were identified in the HP group compared to the LP group (Supplementary Table S12). The co-expression and colocalization analyses of lncRNAs and mRNAs were used to identify trans and cis interactions between lncRNAs and mRNAs. A total of 1540 lncRNA and 1515 mRNA target genes were found, respectively. These 1512 genes were considered trans-interaction target genes of DElncRNAs (Supplementary Table S13). The genomic location analysis showed that one DElncRNAs was located near three mRNAs, which was considered to demonstrate cis interactions of the DElncRNAs (Supplementary Table S14). A total of three DEmRNAs were the lncRNAs target genes and they were trans-acted by lncRNAs.

### 3.5. CeRNA Regulatory Network

**Figure 5:**
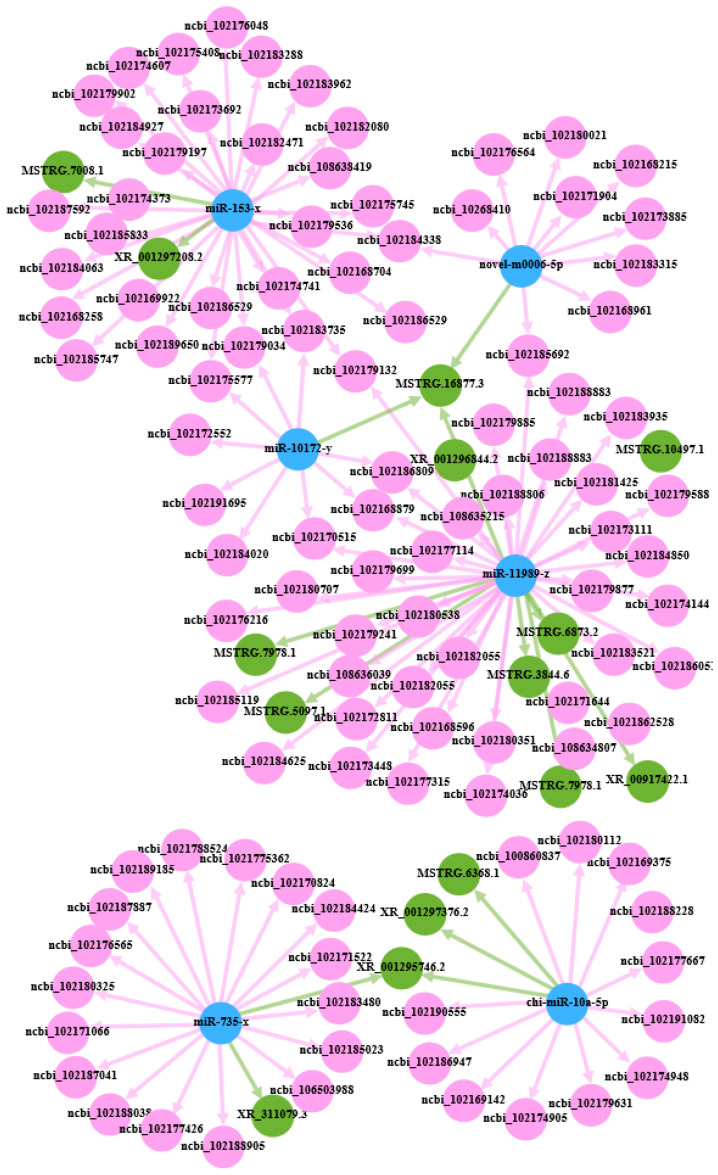
Predicted competing endogenous RNA (ceRNA) networks.

**Figure 6:**
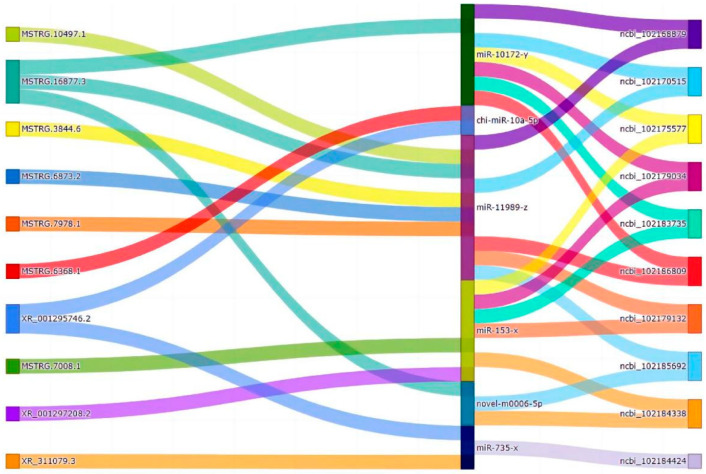
Sankey diagram showing the relationship between the top 10 mRNAs and lncRNAs with connectivity and miRNA-targeted regulation.

To obtain the competing relationships, we predicted the possibilities for differentially expressed miRNAs to bind to DEcircRNAs/DEmRNAs/DElncRNAs. Based on the ceRNA hypothesis, lncRNAs could act as a sponge for miRNA, regulating the availability of miRNAs for binding to mRNA targets [20]. The ceRNA network was constructed using DEmiRNA target genes (DETmiRNAs), DEmRNAs, DElncRNAs, and DEcircRNAs. The networks were constructed as follows: (1) the detection of the negatively correlated DEmRNA–DETmiRNAs pairs (Supplementary Table S15); (2) the detection of the negatively correlated DElncRNA–DETmiRNA pairs (Supplementary Table S16); and (3) the detection of the negatively correlated DEcircRNA–DETmiRNA pairs (Supplementary Table S17). Of those, the DEmRNA–DETmiRNA and DElncRNA–DETmiRNA pairs shared the same miRNA response elements (MREs). After filtering redundant interactions and correlation filtering, 106 DEmRNAs and 14 DElncRNAs were predicted to be the targets of six DEmiRNAs. Subsequently, a hypergeometric cumulative distribution function test was conducted to identify RNA pairs competing for miRNA binding. For a total of 323 pairs, the ceRNA pair with a hypergeometric test value of less than 0.05 was selected as the final ceRNA pair (Figure 5). In the network, there were a few hub nodes with high connectivity, referred to as hub genes. We then analyzed the protein–protein functional interaction (PPI) network using Cytoscape software to visualize the PPI using the MCC algorithm in the CytoHuba plugin to select the top 10 hub genes (Supplementary Table S18). A Sankey diagram of the relationship between the top 10 mRNAs and lncRNAs with connectivity and miRNA-targeted regulation was drawn, as shown in Figure 6.

### 3.6. qPCR Validation of Differentially Expressed mRNAs

**Figure 7:**
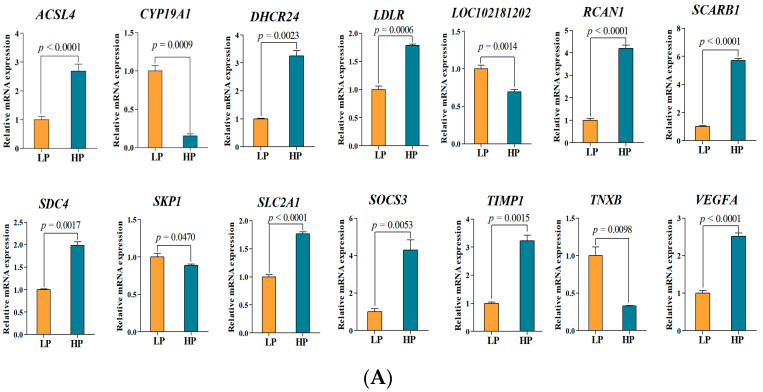

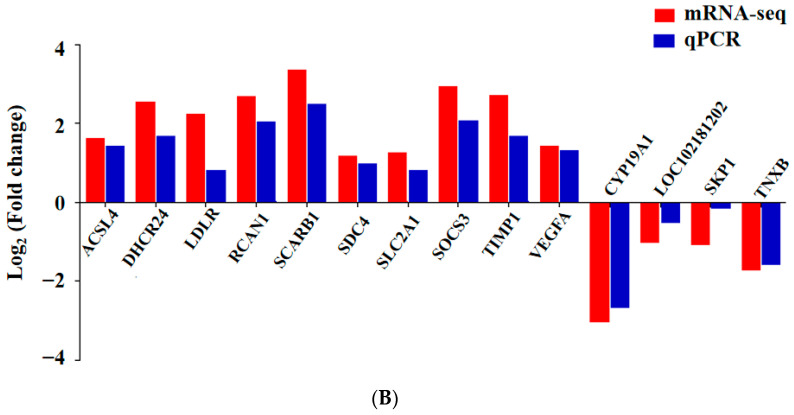
Verification of DEmRNAs via qPCR. (**A**) qPCR verification of 14 randomly selected DEmRNAs. Data represent the mean ± SD. (**B**) Comparison of mRNA sequences and quantitative PCR results based on log2 (fold change) expression.

In order to validate the findings of the RNA-seq analysis, 14 DEmRNAs were randomly selected for the qPCR analysis. As shown in Figure 7A, the expression of all of the selected mRNAs was significantly different between the two groups (*p* < 0.01). Furthermore, the qPCR results showed the same tendencies (upregulation or downregulation) as those derived from the RNA sequencing data (Figure 7B). This indicated that our transcriptome data were credible.

## 4. Discussion

This study examined the expression of mRNA and non-coding RNAs in ovarian tissues, including lncRNAs, circRNAs, and miRNAs, between high- and low-fecundity groups in Chongming white goats. A total of 724 differentially expressed mRNAs, 112 differentially expressed lncRNAs, 10 differentially expressed miRNAs, and 178 differentially expressed circRNAs were screened out. Collectively, the high expression of non-coding RNA and mRNA indicated the subtle regulation of maintaining homeostasis in the ovaries during estrus. Through the bioinformatics analysis, we obtained the following key molecular markers: (ncbi_102168879 (SEC14L5), ncbi_102183735 (IL17RE), ncbi_102184338 (GABRB2), ncbi_102179034 (FAT4), ncbi_102185692 (ADSSL1), ncbi_102170515 (MLLT3), ncbi_102186809 (LYPD6), ncbi_102175577 (FRMD3), ncbi_102184424 (ADGRB1), miR-10172-y, chi-miR-10a-5p, miR-11989-z, miR-153-x, and miR-735-x).

SEC14L5, belonging to the subgroup of SEC14-containing proteins, was found to be significantly altered in terms of body size and lipid transport and metabolism, which may have resulted from selection driven by a demand for energy-rich food during estrus in goats [21]. As is well known, IL17RE has important roles in the function and activation of immune cells. However, IL17RE is also a novel growth receptor-like molecule that is capable of supporting cellular mitogenesis through the RAS/MAPK pathway [22]. It is worth noting that follicle development in females is dependent upon a properly regulated RAS/MAPK signaling pathway [23]. In addition, it has been reported that ADGRB1 activates the Rho–ROCK pathway and thereby regulates follicle development [24]. GABRB2 is a component of the heterodimeric G-protein-coupled receptor for GABA (gamma-aminobutyric acid), which mediates the bulk of rapid inhibitory neurotransmissions in the adult mammalian central nervous system. Multiple species have been studied in vivo for their effects on the secretion of gonadotropins through the manipulation of the GABAA receptor, while GABA inhibits the reproductive axis [25]. Additionally, previous studies have shown that GABRB2 transcription is regulated by epigenetic mechanisms, such as methylation [26]. In addition, LYPD6 enriched in cortical GABAergic populations has been found to effectively regulate nAChR signaling in GABAergic interneurons [27]. As a protocadherin, FAT4 has been shown to be involved in the Hippo signaling pathway and planar cell polarity (PCP) in vitro and in vivo [28]. The Hippo signaling pathway is an evolutionarily conserved pathway and is involved in virtually all stages of ovarian development and function, including estrogen and progesterone synthesis, follicular growth, and oocyte maturation [29]. ADSS1 is a key enzyme for guanine nucleotide de novo synthesis and is involved in converting IMP (inosinmonophosphat) to GMP; the cyclic form of GMP was shown to be associated with preparing the oocyte for fertilization and reproductive success [30]. MLLT3 functions as a transcription activator, elongates the transcription of target genes, and remodels chromatin in embryogenesis [31]. MLLT3 has also been reported to play essential roles in embryonic development, for instance, modifying histone H3K79 to affect cerebral cortex development [32]. FRMD3 was identified as one of the cytoskeletal proteins. The contributions of the cytoskeletal proteins to the emergence of polarity during oocyte maturation are noteworthy [33]. During oocyte maturation, the cytoskeletal proteins also orchestrate the rearrangement of organelles in preparation for fertilization [34]. Therefore, these play key roles in ovary development, and their differential expression levels in goat may contribute to the increased fecundity rate.

Increasing evidence shows that miRNAs play an important regulatory role in the growth and development of oocytes. Among the differentially expressed miRNAs in this experiment, we found five DEmiRNAs (miR-10172-y, chi-miR-10a-5p, miR-11989-z, miR-153-x, and miR-735-x) based on the circRNA–miRNA–mRNA network that had a significant relationship with mammalian fertility. It is worth noting that miR-10a is associated with granulosa cell proliferation, steroid hormone synthesis, and ovary apoptosis regulation [35]. On the other hand, the target genes of DEmiRNAs were further analyzed via GO and KEGG pathway enrichment analyses. The GO functional enrichment analysis revealed that the target genes of DEmiRNAs participated mainly in cellular processes, biological regulation, developmental processes, biological adhesion, and reproduction. This result proved that many target genes of DEmiRNA, such as KITLG, CYP19A1, and GHRHR, were involved in the regulation of a variety of reproductive activities. As a strong candidate gene affecting litter size in goats, KITLG mRNA expression in ovarian follicles was localized to granulosa cells in all species, which is thought to have many roles in ovarian function [36]. It is known that CYP19A1 is widely expressed in ovaries and granulosa cells, where it converts androgens into estrogens. Importantly, CYP19A1 has been demonstrated to influence lambing traits in goats by affecting granulosa cell proliferation, hormone secretion, and the expression of candidate genes associated with traits for multiple births [10,37]. GHRHR, a member of the superfamily of the G-protein-coupled receptor subfamily, works together with the growth-hormone-releasing hormone (GHRH) to regulate the growth hormone axis and the development and proliferation of pituitary somatotropes. However, its roles in goat fecundity have not been reported yet. The KEGG enrichment analysis showed that the Wnt signaling pathway, signaling pathways regulating the pluripotency of stem cells, the AMPK signaling pathway, cell adhesion molecules (CAMs), and MAPK signaling pathway were highly enriched, further demonstrating the possibility of those pathways regulating reproductive processes. The Wnt signaling pathway regulates a number of cellular processes, including differentiation, cell proliferation, and stem cell pluripotency [38]. Furthermore, signaling pathways regulating the pluripotency of stem cells are the key pathways in embryo development [39]. In addition, we compared the analysis of miRNA and mRNA expression profiles of high- and low-fertility ovaries of black goats with the work of Liang et al., and found that chi-miR-122 showed high expression in both sets of high-fertility goats [13]. This tendency showed the same trend as that seen in our results. Similarly, Wang et al. (2018) also reported miR-122 to be a candidate miRNA involved in the effects of monochromatic light on pigeon reproduction [40]. Therefore, the upregulation of chi-miR-122 may be a useful strategy to increase ovulation rates in goats.

Then, we conducted further research on DEmRNAs and DElncRNAs. The GO enrichment analysis showed that DEmRNAs were mainly involved in developmental processes, the negative regulation of biological processes, cellular processes, cellular component organization or biogenesis, reproductive processes, reproduction, and biological regulation. We speculate that these signal pathways may be involved in reproduction traits. The KEGG analysis revealed that most of the DEmRNAs were annotated in the synthesis and secretion of cortisol and steroids. Cortisol and steroid hormones are known to regulate ovarian cell proliferation, apoptosis, folliculogenesis, and folliculogenesis [41]. Animal multifertility is closely related to the occurrence and development of follicles, especially directly related to the number of ovulatory dominant follicles [42]. In another study on the regulation of follicular development in black goats, it was also found that the conversion of progesterone and testosterone occurs in the process of ovarian steroidogenesis, which is one of the most important hormonal pathways known for follicular maturation [10]. In addition, the PI3K–AKT signaling pathway was another significant pathway found in the DEmRNAs of enriched genes in the KEGG enrichment analysis. Indeed, PI3K–AKT has been shown to be involved in multiple cellular functions, such as cell cycle progression, cell proliferation, differentiation, migration, etc. [43]. The PI3K–AKT pathway is also recognized as an integrator of reproductive functions [44]. In this study, 28 DEmRNAs (including FGF23, VEGFA, NOS3, TNXB, EPHA2, etc.) were located in the PI3K–AKT pathway. Similarly, and in agreement with the transcriptomics results, the expression of VEGFA mRNAs was significantly higher in the HP group compared to the LP group when using qPCR, and the expression of TNXB mRNAs was significantly lower in the HP group compared to the LP group. The VEGFA complex plays an important role in the development of angiogenesis, skeletal growth, and ovarian angiogenesis. A previous study on perinatal rat ovaries reported that VEGFA can affect primordial follicle activation through vascular-dependent mechanisms and alter primary follicle counts through vascular-independent mechanisms [45]. Another study identified that in human ovaries, the mechanism of angiogenesis and vessel development may be associated with the downregulation of TNXB [46]. Next, we found three DEmRNAs (LOC102174738, SLC2A1, and RASAL1), which were the lncRNA target genes, and they were trans-acted by lncRNAs. Then, we annotated the DElncRNA target genes to the KEGG databases. The KEGG enrichment analysis showed that oocyte meiosis, TGF-beta signaling pathway, Hedgehog signaling pathway, Wnt signaling pathway, and RAS signaling pathway were highly enriched. These pathways are strongly correlated with mammalian development, including prolificacy; therefore, they could potentially be involved in the regulation of goat fecundity.

CircRNA is implicated in transcriptional and post-transcriptional gene regulation in eukaryotic cells. It interacts with RNA-binding proteins to regulate the expression of targeted genes. Due to their unique structure, CircRNAs can have special biological functions. To further explore the potential functions of circRNAs in goat ovarian tissues between high- and low-fecundity in Chongming white goats, we annotated the DEcircRNAs target genes to GO and KEGG to identify the molecules and pathways regulated by these DEcircRNAs. The GO functional enrichment analysis revealed that the circRNA source genes participated mainly in cellular component organization or biogenesis, the rhythmic process, developmental process, biological regulation, reproductive process, and reproduction. This result proved that many host genes of DEcircRNAs, such as BMPR1B, TGFB2, and KIT, were involved in the regulation of a variety of reproductive activities. As the first major prolificacy gene discovered in sheep, BMPR1B (FecB) expression in the follicles of multiparous Hu sheep was significantly higher than that of uniparous Hu sheep, which indicated that it may influence the ovulation rate by regulating the BMP/Smad signal pathway [47]. Similarly, it has also been reported that BMPR1B is involved in the regulation of fecundity in goats, which indicates that it plays an important part in cumulus cell expansion, ovulation cycle, follicular development, and maturity [48]. TGFB2 is known as an isoform of the TGFB family, and the TGFB family has been shown to be important for ovary function at a number of developmental stages [49]. The prosurvival factor, KIT, and its receptor have been proven to affect germ cell proliferation and migration and the primordial to primary follicle transition [50]. Through the KEGG pathway analysis, we determined that these DEcircRNA target genes were enriched in pathways, including the Rap1 signaling pathway, MAPK signaling pathway, Hippo signaling pathway, and Hedgehog signaling pathway. Since the Rap1 signaling pathway is involved in the MAPK pathway, the latter should play diverse roles in reproductive tissues [51]. The role of the MAPK signaling pathway in female reproductive regulation was already discussed above. The interaction between the Hippo and Hedgehog pathways was demonstrated in mammals. A previous study reported that the Hippo pathway acts downstream of the Hedgehog signaling pathway to regulate follicle stem cell maintenance in the Drosophila ovary [52]. As a result of these stem cells, the primary follicular resource can be produced during reproduction, and the development of the follicle determines the ovulation rate.

## 5. Conclusions

In this study, a dataset of goat ovary miRNA, circRNA, lncRNA, and mRNA profiles was developed, as well as profiles showing differential expression between high and low fecundity in the ovaries of Chongming white goats during estrus. As a result of our data, we built networks among differentially expressed miRNAs, circRNAs, lncRNAs, and mRNAs, shedding light on the regulation of goat fecundity genes. Collectively, our study indicated that the transcriptomic analysis of goat ovaries can provide novel insights into the fecundity of goats, implying the presence of numerous previously uncharacterized elements in developmental biology.

## Figures and Tables

**Table 1:** Primer sequences used for qPCR in this study.

Gene Name	GenBank Accession No.	Primer Sequences (5-3)	Tm (°C)	Length (bp)
*SCARB1*	XM_018061401.1	F: GGCACAAGAGCAACATCACC	59	145
		R: ATCGACGCACTCAAGACCAG		
*CYP19A1*	NM_001285747.1	F: GCATCTGGACAGGTTGGAGG	61	344
		R: CAAAAATCAACTCAGTGGCGAA		
*LDLR*	XM_005682375.3	F: CGCCTACCTCTTCTTCACCAA	60	105
		R: TCCAGGGCAACCACATTCTT		
*SDC4*	XM_018057920.1	F: TGCACCCCTTGGTCCCTAT	60	203
		R: TGCCAGCACCTCTGTCCTCT		
*LOC102181202*	XM_018038833.1	F: CTGTGATCTGGAGGAAGAAGC	61	78
		R: CCTGGGCACTGTCATTGTT		
*DHCR24*	XM_005678362.3	F: TGAAGACAAACCGAGAGGGC	60	133
		R: CCAAAGAGGTAGCGGAAGATG		
*TNXB*	XM_018038915.1	F: ATACACAGACGAGGATGGGCA	60	217
		R: CGGGGTTTGGGTGGTAGAGA		
*SKP1*	XM_018050387.1	F: TTGGAAGATTTGGGAATGGAT	59	208
		R: GTGTCCCTTGGTCAACTTTCAG		
*VEGFA*	XM_018038496.1	F: GGGGCTGCTGTAATGACGA	60	97
		R: TGCTGGCTTTGGTGAGGTTT		
*TIMP1*	XM_005700836.3	F: CACAGGTCCCAGAATCGCA	60	159
		R: TTCCTCACAACCAGCAGCATA		
*SOCS3*	XM_018063683.1	F: CGAGAAGATCCCTCTGGTGTT	59	107
		R: CTTTCTCGTAGGAGTCCAGGTG		
*ACSL4*	XM_018044177.1	F: AGTCCATATCGTTCTGTCACGC	61	123
		R: TCCCAGGCTCTCCTTCTTCC		
*SLC2A1*	NM_001314223.1	F: CGGCAGATGATGCGAGAGA	62	229
		R: GCGACACGACAGTGAAGGCT		
*RCAN1*	XM_018055819.1	F: GCTTCAAACGGGTCAGAATAAA	59	140
		R: CAGGTGCGAACTGCCTATGT		
*RPL19*	XM_005693740.3	F: ATCGCCAATGCCAACTC	60	154
		R: CCTTTCGCTTACCTATACC		

## Data Availability

Raw sequence data were uploaded in the China National Center for Bioinformation (CNCB; https://www.cncb.ac.cn, accessed on 28 February 2024) with accession number: PRJCA023977.

## Supplementary Materials

The following supporting information can be downloaded at https://www.mdpi.com/2076-2615/14/7/988/s1: Table S1: Identified circRNAs and their information. Table S2: Differentially expressed CircRNAs between LP and HP groups. Table S3: Enriched GO terms for differentially expressed circRNA source genes. Table S4: Enriched KEGG pathways for differentially expressed circRNA source genes. Table S5: The sequence and expression of identified miRNAs. Table S6: Differentially expressed miRNAs between LP and HP groups. Table S7: Enriched GO terms for target genes of differentially expressed miRNAs. Table S8: Enriched KEGG pathways for target genes of differentially expressed miRNAs. Table S9: Differentially expressed mRNAs between LP and HP groups. Table S10: Enriched GO terms for differentially expressed mRNAs. Table S11: Enriched KEGG pathways for differentially expressed mRNAs. Table S12: Differentially expressed lncRNAs between LP and HP groups. Table S13: Predicted trans-interaction target genes of differentially expressed lncRNAs. Table S14: Predicted cis-interaction target genes of differentially expressed lncRNAs. Table S15: Negatively correlated DEmRNA–DETmiRNA pairs. Table S16: Negatively correlated DElncRNA–DETmiRNA pairs. Table S17: Negatively correlated DEcircRNA–DETmiRNA pairs. Table S18: The top 10 mRNAs and lncRNAs with connectivity and miRNA-targeted regulation.
